# Effect of Opioid-Sparing Multimodal Anesthesia Within an Enhanced Recovery After Surgery (ERAS) Protocol on Postoperative Recovery in Gynecologic Oncology Surgery: A Randomized Clinical Study

**DOI:** 10.7759/cureus.104812

**Published:** 2026-03-07

**Authors:** Maria Bourazani, Nikolaos Fyrfiris, Petros Galanis, Chrysseida Maglari, Antonios Anagnostopoulos, Dimitrios C Papatheodorou, Martha Kelesi, Sofia Poulopoulou

**Affiliations:** 1 Department of Anaesthesiology, Hellenic Anticancer Institute, Saint Savvas Hospital, Athens, GRC; 2 Clinical Epidemiology Laboratory, Faculty of Nursing, National and Kapodistrian University of Athens, Athens, GRC; 3 Department of Gynecologic Oncology, Hellenic Anticancer Institute, Saint Savvas Hospital, Athens, GRC; 4 Department of Nursing, University of West Attica, Athens, GRC

**Keywords:** eras, eras protocols, gynecologic oncology, gynecologic oncology surgery, multimodal analgesia, opioid-sparing analgesia, postoperative recovery

## Abstract

Background. Major gynecologic oncological procedures are associated with moderate to severe postoperative pain and significant morbidity. Opioids remain a cornerstone of postoperative analgesia but are frequently linked with adverse effects that may delay recovery. Enhanced Recovery After Surgery (ERAS) protocols emphasize multimodal analgesia strategies aimed at minimizing opioid use while improving recovery outcomes. The aim of this study was to evaluate whether an opioid-sparing multimodal anesthesia protocol within an ERAS pathway improves postoperative outcomes in patients undergoing gynecologic oncologic surgery.

Methods. This randomized clinical study included 101 women undergoing major gynecologic oncological procedures. Fifty-one patients were allocated to an ERAS protocol incorporating multimodal anesthesia and opioid-sparing strategies, while 50 patients received conventional perioperative care. Standardized anesthesia management was applied in both groups, with the ERAS group receiving additional multimodal interventions including perioperative magnesium, structured antiemetic prophylaxis, early oral intake, and early transition to oral analgesia. Pain scores, rescue analgesic use, postoperative complications, time to oral intake, gastrointestinal recovery, and discontinuation of intravenous therapy were analyzed.

Results. Patients managed under the ERAS protocol demonstrated significantly lower postoperative pain scores at all measured time points compared with controls (p < 0.001). Rescue analgesic requirements were significantly reduced in the ERAS group (p < 0.001). Early oral feeding within six hours occurred significantly more frequently in the ERAS group than in the control group (p < 0.001). Gastrointestinal recovery and early discontinuation of intravenous therapy were also significantly improved in the ERAS group (p < 0.001). Additionally, ERAS patients exhibited lower incidences of vomiting, sedation, and somnolence postoperatively (p = 0.01, p = 0.01, and p = 0.001, respectively).

Conclusions. Implementation of an opioid-sparing multimodal anesthesia strategy within an ERAS pathway significantly improves postoperative recovery in gynecologic oncologic surgery. The protocol reduces opioid-related adverse effects, enhances gastrointestinal recovery, and supports earlier mobilization and oral intake. These findings reinforce the role of multimodal anesthesia as a key component of ERAS programs in oncological surgery.

## Introduction

Major gynecologic oncological surgeries are frequently associated with moderate to severe postoperative pain, delayed mobilization, and prolonged hospital stay. Opioid analgesics have traditionally been central to perioperative pain management; however, their use is commonly accompanied by adverse effects, including nausea, vomiting, sedation, respiratory depression, and delayed recovery [1-4].

In recent years, perioperative medicine has increasingly focused on opioid-sparing strategies aimed at improving recovery outcomes while minimizing pharmacologic complications. Multimodal anesthesia utilizes combinations of analgesic agents and techniques with different mechanisms of action targeting the nociceptive pathway [5,6].

Enhanced Recovery After Surgery (ERAS) programs integrate evidence-based perioperative practices designed to optimize physiologic stability, reduce surgical stress, and accelerate postoperative recovery [7,8]. Within these pathways, anesthetic management plays a crucial role in determining postoperative outcomes, particularly regarding pain control, gastrointestinal function, and early mobilization [9].

Despite growing international adoption of ERAS protocols, limited data exist regarding their anesthetic implementation in gynecologic oncologic surgery, particularly in European clinical settings. The present randomized clinical study aimed to evaluate whether an opioid-sparing multimodal anesthesia protocol embedded within an ERAS pathway improves postoperative recovery compared with conventional perioperative care in patients undergoing major gynecologic oncological procedures.

The primary aim of this study was to evaluate whether an opioid-sparing multimodal anesthesia protocol within an ERAS pathway improves postoperative outcomes in patients undergoing gynecologic oncologic surgery. The specific objectives were as follows: 1. To record postoperative pain intensity and evaluate the effectiveness of analgesic management; 2. To assess the need for supplemental (rescue) analgesia; 3. To evaluate opioid-related adverse effects, including sedation and somnolence; 4. To record postoperative gastrointestinal function, including oral intake, nausea, vomiting, and bowel activity.

## Materials and methods

Study design and population

This study was a prospective randomized clinical trial conducted as part of a doctoral research project at the gynecologic oncology department of Saint Savvas Anticancer-Oncology Hospital, a tertiary referral oncologic center in Athens, Greece. 

Eligible patients with malignancies of the internal genital organs undergoing major gynecologic oncologic surgery were randomly assigned to two groups: Group A followed the ERAS protocol, while Group B received conventional postoperative care.

Patients were randomly assigned to the ERAS or conventional care group using a computer-generated randomization sequence. Due to the nature of the intervention, anesthesiologists and surgical teams were aware of group allocation. Allocation concealment was ensured using sequentially numbered opaque sealed envelopes prepared by an independent investigator. However, postoperative outcome assessment, including pain scoring and complication recording, was performed by investigators blinded to treatment group in order to reduce assessment bias.

Eligibility criteria

Inclusion Criteria

The inclusion criteria were as follows: age greater than 18 years; ability to understand and communicate in Greek; absence of cognitive impairment

Exclusion Criteria

The exclusion criteria were as follows: current treatment for chronic pain or antidepressant use; acute or chronic renal or hepatic disease; chronic mobility impairment

The sample size was informed by preliminary institutional data suggesting detectable clinically meaningful differences between groups; therefore, the final cohort of 101 patients was considered adequate for the study objectives. Data collection was performed between 2020 and 2024.

This manuscript represents a focused secondary analysis of anesthetic and analgesic outcomes derived from the same randomized trial population previously reported for overall ERAS recovery outcomes, addressing a distinct research question and set of endpoints [10].

Standardized multimodal anesthesia protocol

Recent studies highlight the importance of multimodal anesthesia in reducing perioperative opioid exposure and improving recovery outcomes [2,3,11,12]. Multimodal general anesthesia is defined as an anesthetic strategy that integrates knowledge of neuroanatomy and neurophysiology to modulate the nociceptive processing and anesthetic depth, enabling rational titration of anesthetic and analgesic agents while minimizing drug-related adverse effects [5,6,13].

Advances in anesthetic monitoring allow reliable assessment of anesthetic depth and neuromuscular blockade using electronic monitoring systems in conjunction with clinical evaluation. The bispectral index (BIS) remains the most widely used method for estimating anesthetic depth and has demonstrated superior performance compared with alternative monitoring techniques. Maintenance of anesthetic depth within a minimum alveolar concentration (MAC) range of 0.7-1.3 and BIS values between 40 and 60 has been associated with reduced risks of intraoperative awareness, delayed emergence, and perioperative complications. Conversely, excessively deep anesthesia (BIS < 45) has been linked to increased mortality risk [14,15]. BIS values may be influenced by neuromuscular blockade, so simultaneous monitoring with a peripheral nerve stimulator was performed in all patients.

Increasing attention has been directed toward the potential impact of anesthetic techniques on postoperative immune modulation and long-term oncological consequences, such as overall survival and cancer recurrence. However, current evidence remains insufficient to support definitive recommendations regarding anesthetic selection in oncological surgery [16,17].

Preoxygenation with an inspired oxygen concentration above 21% was routinely performed prior to induction to prevent tissue hypoxia during airway management. Maintenance of adequate oxygenation throughout anesthesia was considered essential, as intraoperative hypoxaemia may impair cellular metabolism and adversely affect outcomes [18].

Normothermia was actively maintained intraoperatively, given that perioperative hypothermia has been shown to prolong neuromuscular blockade, increase bleeding risk, and contribute to cardiovascular complications, impaired wound healing, and postoperative infections [19].

At the end of surgery, complete reversal of neuromuscular blockade was ensured to avoid residual paralysis and respiratory complications. Sugammadex was used for the reversal of rocuronium-induced blockade, allowing rapid recovery independent of blockade depth. Extubation was performed only after achieving a train-of-four ratio ≥ 0.9 to confirm adequate neuromuscular function [20].

ERAS anesthetic pathway implementation

The ERAS pathway was initiated one day prior to surgery with patient-informed consent, education, perioperative planning, and clarification of therapeutic goals [21,22].

Patients in the ERAS group were permitted solid food up to six hours and carbohydrate-containing fluids up to four hours before anesthesia unless contraindicated. In contrast, patients in the conventional group adhered to the traditional six-hour fasting protocol and received 1 liter of preoperative intravenous hydration with Ringer’s lactate [21,23-25].

Upon arrival in the operating room, standard monitoring was applied, including continuous electrocardiography, noninvasive blood pressure monitoring, and pulse oximetry. A peripheral venous catheter (16-20G) was inserted, and preoxygenation was performed.

Induction of anesthesia consisted of fentanyl 2.5 mcg/kg, propofol 2-2.5 mg/kg, and rocuronium 0.6 mg/kg to facilitate tracheal intubation. Anesthesia was maintained using a fresh gas flow of 2 L/min with an oxygen/air mixture, and desflurane was titrated to maintain a MAC of approximately 1.0 [15]. Additional fentanyl was administered according to hemodynamic responses, while neuromuscular blockade was guided by peripheral nerve stimulation monitoring [26].

Multimodal analgesia was initiated immediately after induction using intravenous paracetamol 1 g and parecoxib 40 mg, combined with morphine 0.1 mg/kg as required. Patients in the ERAS group additionally received intraoperative magnesium sulfate 50 mg/kg as part of the opioid-sparing strategy [27-29].

Patients at increased risk of postoperative nausea and vomiting received standardized prophylaxis with ondansetron 4 mg and metoclopramide 10 mg (maximum total daily dose: 20 mg), with dexamethasone 4 mg administered as rescue therapy when required [30-33].

Throughout the procedure, physiologic homeostasis was maintained through monitoring of glycemia, fluid balance, urine output, and temperature [24,34,35]. Active warming devices and warmed intravenous fluids were used to maintain core temperature near 36°C [19].

Neuromuscular blockade reversal with sugammadex (2 mg/kg) was performed in both groups before extubation [20]. Patients were then transferred to the recovery unit (post-anesthesia care unit) for approximately one hour prior to returning to the ward.

Postoperatively, ERAS patients continued multimodal analgesia and antiemetic prophylaxis initiated intraoperatively. Oral intake was introduced within four to six hours, provided nausea was absent, and intravenous therapy was discontinued once oral hydration was adequate. On postoperative day one (POD 1), analgesia was transitioned to oral multimodal therapy, whereas conventional care patients followed traditional fasting and intravenous analgesia protocols [36].

The detailed anesthetic, analgesic, and antiemetic components of the ERAS protocol compared with conventional care are presented in Table 1.

**Table 1:** Perioperative anesthetic and analgesic interventions in the ERAS and conventional care groups. This table summarizes the standardized perioperative anesthetic, analgesic, and antiemetic components applied in each study group according to the treatment protocol. ERAS: Enhanced Recovery After Surgery; PO: per oral; PONV: postoperative nausea and vomiting; PRN: as needed; POD: postoperative day

Phase	ERAS Recovery Protocol	Conventional Postoperative Care
Day before surgery	Patient education about the ERAS program and informed consent	Informed consent about the anesthesia and surgery
	Therapeutic planning, goal setting, and patient participation	
	Patients were allowed clear fluids up to two to four hours and solid food up to six hours before surgery. Carbohydrate drinks permitted (200 ml up to 4h or 50 ml up to 2h preoperatively).	Nil PO, Six hours traditional fasting. Maintenance IV fluids (Ringer’s lactate 1000 ml).
Intraoperative Period	Standardized anesthesia protocol with maintenance of normothermia, normoglycemia, and euvolemia	Standardized anesthesia protocol
	Parecoxib 40 mg IV, paracetamol 1000 mg IV, morphine 0.1 mg/kg IV, magnesium sulfate 50 mg/kg IV (single dose)	Parecoxib 40 mg IV, paracetamol 1000 mg IV, morphine 0.1 mg/kg IV
	PONV IV prophylaxis protocol with ondansetron 4 mg, metoclopramide 10-20 mg/24h, and dexamethasone 4 mg if needed.	Ondansetron 4 mg and metoclopramide 10-20 mg IV, if indicated
POD 0	Paracetamol 1000 mg ×4, parecoxib 40 mg ×2, morphine 0.1 mg/kg IV	Paracetamol 1000 mg ×4 with tramadol 50–100 mg ×4 or morphine 0.1 mg/kg IV
	PONV protocol	Ondansetron 4 mg ×2 and metoclopramide if indicated
	IV fluids until oral intake is established	IV fluids 2-3 L/day
	Oral fluids within four to six hours; clear diet and jelly allowed	—
POD 1	Oxycodone 5 mg/paracetamol 325 mg PO every four to six hours PRN; lornoxicam 8 mg twice daily PO	Paracetamol 1000 mg + tramadol 50-100 mg IV
PODs 2–3	Same oral regimen (oxycodone/paracetamol + lornoxicam)	Paracetamol 1000 mg + tramadol 50–100 mg IV
Discharge	Oxycodone 5 mg /paracetamol 325 mg PO q: 4-6 PRN and lornoxicam 8 mg PO twice daily	Oral regimen: paracetamol 400 mg/codeine 10 mg/caffeine 50 mg PO + rescue paracetamol 1 gr PO daily
	Telephone follow-up for three days and reassessment at POD7 or POD15	Reassessment on POD 15

Outcomes and statistical analysis

Primary outcomes included postoperative pain scores and opioid requirements. Secondary outcomes included rescue analgesic use, postoperative nausea and vomiting, timing of oral feeding, gastrointestinal recovery, discontinuation of intravenous therapy, and postoperative complications.

Categorical variables were presented as absolute and relative frequencies, while continuous variables were expressed as mean, standard deviation, median, minimum, and maximum values. Normality of continuous variables was assessed using the Kolmogorov-Smirnov test.

To evaluate baseline comparability between the ERAS and conventional care groups, the following statistical tests were applied: chi-square test for associations between categorical variables; Fisher’s exact test, when expected frequencies were small; chi-square test for trends, for associations involving ordinal variables; Independent samples t-test for normally distributed continuous variables; Mann-Whitney U test for non-normally distributed continuous variables.

No statistically significant differences were observed between groups regarding demographics, diagnosis, surgical procedure, medical history, or laboratory values, indicating that randomization successfully minimized confounding effects. Therefore, multivariable analyses were not considered necessary.

Changes in pain scores over time were analyzed using repeated-measures analysis of variance. A two-sided significance level of a = 0.05 was applied throughout. Statistical analyses were performed using IBM SPSS Statistics version 21.0 (IBM Corp., Armonk, NY, USA).

Postoperative pain intensity was assessed using the Numeric Rating Scale (NRS), where 0 represents no pain, and 10 represents the worst imaginable pain at postoperative hours 0, 2, 6, 12, 18, and 24 [37]. Assessments were performed at predefined postoperative time points by trained personnel using a standardized protocol.

Clinical data were collected from anesthesia charts, operative records, medical files, and patient interviews.

## Results

Baseline characteristics

A total of 101 women undergoing major gynecologic oncologic surgery were included in the study. Fifty-one patients (50.5%) were randomized to the ERAS group, while 50 patients (49.5%) were allocated to the conventional care group. The sample size was informed by preliminary institutional data, which suggested that clinically meaningful differences could be detected within the enrolled population [10].

No statistically significant differences were observed between the two groups regarding demographic characteristics, diagnosis, type of surgery, comorbidities, or preoperative laboratory values, confirming the comparability of the study population. Table 2 demonstrates the baseline demographic and clinical characteristics. 

**Table 2:** Baseline demographic and clinical characteristics Data are presented as mean ± standard deviation (SD) for continuous variables and n (%) for categorical variables. Continuous variables are presented as mean ± SD and were compared using the independent samples t-test. Categorical variables are presented as n (%) and were compared using the chi-square (χ²) test or Fisher’s exact test when expected cell counts were small. Test statistic values are reported for each comparison. Statistical significance was defined as p < 0.05.

Variable	Control n=50	ERAS n=51	Test	Statistic	p-value
Age (years)	56.7 ± 11.1	52.8 ± 12.5	t-test	t = 1.66	0.10
BMI (kg/m^2^)	26.8 ± 4.8	26.4 ± 5.2	t-test	t = 0.76	0.70
Smoking (n, %)	22 (44%)	18 (35.3%)	Fisher’s exact	—	0.40
Alcohol (n, %)	9 (18%)	6 (11.8%)	Fisher’s exact	—	0.40
Endocrine (n, %)	21 (42%)	20 (39.2%)	χ² test	χ² = 0.04	0.80
Cardiovascular (n, %)	15 (30%)	12 (23.5%)	χ² test	χ² = 0.45	0.50
Respiratory (n, %)	5 (10%)	6 (11.8%)	Fisher’s exact	—	0.80
Allergy (n, %)	9 (18%)	12 (23.5%)	χ² test	χ² = 0.54	0.50
Previous malignancy (n, %)	6 (12%)	13 (25.5%)	Fisher’s exact	—	0.10

The mean age was 56.7 years in the control group and 52.8 years in the ERAS group; age did not differ between groups (p=0.10). The mean BMI was 26.8 kg/m² in the control group and 26.4 kg/m² in the ERAS group (p = 0.70).

Smoking was reported in 22 (44%) patients of the control group and 18 (35.3%) patients of the ERAS group (p = 0.40), while alcohol consumption was reported in nine (18%) patients and six (11.8%), respectively (p = 0.40). The most common comorbidities in both groups included endocrine disorders, cardiovascular disease, allergies, respiratory disease, and prior malignancy, with no statistically significant differences between the two groups. 

Preoperative laboratory parameters were comparable between groups (Table 3). Mean hemoglobin was 12.6 g/dL in the control group and 12.7 g/dL in the ERAS group (p = 0.70). Mean hematocrit values were 38.7% and 38.9%, respectively (p = 0.80). Mean white blood cell counts were 7.6 ×10⁹/L and 6.9 ×10⁹/L, respectively (p = 0.10), and mean glucose levels were 101 mg/dL and 96.7 mg/dL (p = 0.30).

**Table 3:** Preoperative laboratory values Data are presented as mean ± standard deviation (SD). Continuous variables are presented as mean ± SD and were compared using the independent samples t-test. Test statistic values are reported for each comparison. Statistical significance was defined as p < 0.05. ERAS: Enhanced Recovery After Surgery

Variable	Control group n=50 (mean ± SD)	ERAS group n=51 (mean ± SD)	Test	Test statistic	p-value
Hemoglobin (g/dL)	12.6 ± 1.5	12.7 ± 1.1	t-test	t = 0.50	0.70
Hematocrit (%)	38.7 ± 4.5	38.9 ± 3.2	t-test	t = 0.38	0.80
White blood cells (×10⁹/L)	7.6 ± 2.1	6.9 ± 2.0	t-test	t = 1.87	0.10
Glucose (mg/dL)	101.0 ± 21.2	96.7 ± 20.9	t-test	t = 1.01	0.30

Postoperative pain trajectory

Postoperative pain scores differed significantly over time according to repeated-measures analysis of variance (p < 0.001). 

In the immediate postoperative period (0 to two hours), the mean pain score was 3.6 in the control group and 3.1 in the ERAS group. Pain intensity increased during the early postoperative period, peaking between six and 12 hours after surgery, where mean scores reached 4.6 in the control group and 3.6 in the ERAS group.

From the first postoperative day onward, pain progressively declined in both groups; however, ERAS patients consistently reported lower pain scores. On POD 1, the mean pain score was 4.4 in the control group versus 2.5 in the ERAS group. By POD 3, mean pain scores were 3.0 and 1.3, respectively, and on the day of discharge, mean scores were 2.1 in controls and 1.0 in ERAS patients. Table 4 shows the postoperative pain scores over time.

**Table 4:** Postoperative pain scores over time Pain intensity is presented as mean ± standard deviation (SD). Comparisons between groups over time were performed using repeated-measures analysis of variance (ANOVA). Statistical significance was defined as p < 0.05. Repeated-measures ANOVA demonstrated a significant difference over time (p < 0.001). POD: postoperative day

Time point	Control mean ± SD	ERAS mean ± SD
0–2 hours	3.6 ± 1.0	3.1 ± 1.2
2–6 hours	4.0 ± 1.2	3.5 ± 1.0
6–12 hours	4.6 ± 1.2	3.6 ± 1.0
POD 1	4.4 ± 1.4	2.5 ± 1.0
POD 2	3.6 ± 1.5	2.0 ± 0.8
POD 3	3.0 ± 1.2	1.3 ± 0.6
Day of discharge	2.1 ± 1.0	1.0 ± 0.5

**Figure 1:**
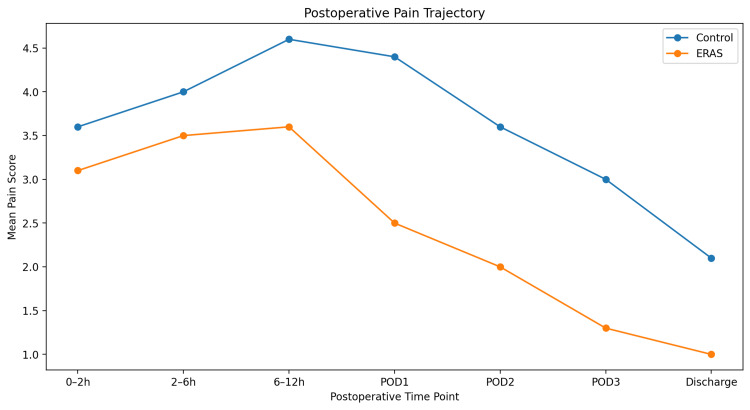
Postoperative pain scores over time Mean pain scores at each postoperative time point were significantly lower in the ERAS group (p < 0.001). ERAS: Enhanced Recovery After Surgery; POD: postoperative day; h: hours

The trajectory of postoperative pain is illustrated in Figure 1.

Analgesic requirements

The number of analgesic administrations was significantly lower in the ERAS group compared with the control group (p < 0.001). Table 5 demonstrates the postoperative analgesic use in two groups. 

**Table 5:** Analgesic consumption Data are presented as mean ± standard deviation (SD), median, and n (%). Continuous variables are presented as mean ± SD and were compared using the independent samples t-test. Categorical variables are presented as n (%) and were compared using the chi-square (χ²) test. Test statistic values are reported for each comparison. Statistical significance was defined as p < 0.05. ERAS: Enhanced Recovery After Surgery

Variable	Control group n=50	ERAS group n=51	Test	Test statistic	p-value
Mean number of analgesic doses ± SD	1.5 ± 1.5	0.3 ± 0.6	t-test	t = 5.31	<0.001
Rescue analgesia required, n (%)	34 (68%)	10 (19.6%)	χ² test	χ² = 24.05	<0.001

The mean number of administered analgesic doses was 1.5 in the conventional group compared with 0.3 in the ERAS group. The median number of doses was one in the control group and 0 in the ERAS group.

Similarly, rescue analgesia was required in 34 (68%) patients of the conventional care group compared with only 10 (19.6%) patients of the ERAS group (p < 0.001).

Early oral intake

Early oral intake within six hours postoperatively occurred in 44/50 (88%) patients of the ERAS group compared with only 2/49 (4.1%) patients of the control group (p < 0.001).

Gastrointestinal recovery

Early restoration of gastrointestinal function occurred in 46 of 50 (92%) patients of the ERAS group compared with eight of 49 (16.3%) patients of the control group (p < 0.001).

Discontinuation of intravenous therapy

Early discontinuation of intravenous therapy was achieved in 41 of 50 (82%) patients of the ERAS group compared with only two of 49 (4.1%) patients of the control group (p < 0.001). Postoperative recovery indicators are presented in Table 6.

**Table 6:** Postoperative recovery indicators Data are presented as n (%). Categorical variables are presented as n (%) and were compared using the chi-square (χ²) test. Test statistic values are reported for each comparison. Statistical significance was defined as p < 0.05. ERAS: Enhanced Recovery After Surgery

Outcome	Control group n=49 (%)	ERAS group n=50 (%)	Test	Test statistic	p-value
Early oral intake	2 (4.1%)	44 (88%)	χ² test	χ² = 70.06	<0.001
Gastrointestinal recovery	8 (16.3%)	46 (92%)	χ² test	χ² = 57.16	<0.001
Early discontinuation of IV therapy	2 (4.1%)	41 (82%)	χ² test	χ² = 61.15	<0.001

Postoperative complications

Postoperative vomiting occurred significantly less frequently in the ERAS group (18%) compared with the control group (36.7%) (p = 0.01). Somnolence occurred in four (8%) patients of the ERAS group compared with 17 (34.7%) patients of the control group (p = 0.001), while sedation occurred in two (4%) and 10 (20.4%) patients, respectively (p = 0.01). No increase in overall postoperative complications was observed, as shown in Table 7.

**Table 7:** Postoperative complications Data are presented as n (%). Categorical variables are presented as n (%) and were compared using the chi-square (χ²) test. Test statistic values are reported for each comparison. Statistical significance was defined as p < 0.05. ERAS: Enhanced Recovery After Surgery

Complication	Control group n=49 (%)	ERAS group n=50 (%)	Test	Test statistic	p-value
Nausea	22 (44.9%)	16 (32%)	χ² test	χ² = 1.74	0.20
Vomiting	18 (36.7%)	9 (18%)	χ² test	χ² = 4.38	0.01
Sedation	10 (20.4%)	2 (4%)	χ² test	χ² = 6.26	0.01
Somnolence	17 (34.7%)	4 (8%)	χ² test	χ² = 6.26	0.001

## Discussion

ERAS programs have transformed perioperative management by integrating evidence-based strategies aimed at minimizing surgical stress and accelerating postoperative recovery [21,22]. Within these pathways, anesthetic management plays a pivotal role, particularly in oncological surgery, where postoperative morbidity may delay further treatment and negatively affect outcomes.

The present randomized clinical study demonstrates that incorporation of opioid-sparing multimodal anesthesia within an ERAS pathway significantly improves postoperative recovery following major gynecologic oncology procedures. Patients managed with the ERAS protocol experienced lower pain scores, reduced need for rescue analgesia, earlier return of gastrointestinal function, and lower rates of opioid-related adverse effects.

The reduction in postoperative pain observed in the ERAS group is consistent with prior studies demonstrating the effectiveness of multimodal anesthesia in limiting opioid exposure and improving analgesic outcomes [1-4]. By exerting different mechanisms of action on the nociceptive circuit, multimodal strategies provide superior analgesia while avoiding the dose-dependent complications associated with opioid monotherapy [11,28].

The significantly lower requirement for rescue analgesia in the ERAS group further supports the efficacy of the multimodal approach. Similar findings have been reported in ERAS-based studies across multiple surgical specialties, where reduced opioid consumption correlates with improved recovery metrics and shorter length of stay [13].

Early restoration of gastrointestinal function and tolerance of oral intake represent central goals of ERAS pathways [25]. In the present study, early feeding was achieved in the vast majority of ERAS patients, whereas it remained rare in the conventional group. This difference likely reflects both improved pain control and the reduced incidence of postoperative nausea and vomiting. The importance of early enteral nutrition in supporting recovery and reducing length of stay has been well documented in ERAS literature [23,24].

Another key finding of this study is the earlier discontinuation of intravenous therapy among ERAS patients. Early transition to oral intake and medications reduces immobilization, promotes independence, and minimizes fluid-related complications. Previous investigations have similarly shown that early oral hydration and medication administration are safe and beneficial components of ERAS protocols [23,24].

Importantly, the ERAS protocol was associated with significantly lower rates of vomiting, sedation, and somnolence, findings consistent with the reduced opioid exposure observed in the intervention group. These outcomes align with previous reports indicating that opioid minimization is strongly associated with fewer postoperative complications and improved patient comfort [21,22].

The present study contributes novel clinical evidence from Greece, where ERAS implementation in gynecologic oncology remains limited. The randomized design and standardized anesthetic management strengthen the validity of the findings and support broader adoption of multimodal anesthesia protocols within ERAS pathways.

Nevertheless, several limitations should be acknowledged. The study was conducted at a single center, which may limit generalizability. Additionally, long-term oncologic outcomes and cost-effectiveness were not evaluated. Future multicenter studies with extended follow-up are needed to confirm these findings and assess their broader clinical implications.

## Conclusions

Implementation of an opioid-sparing multimodal anesthesia strategy within an ERAS pathway significantly improves postoperative recovery in patients undergoing major gynecologic oncologic surgery.

The protocol was associated with reduced postoperative pain, decreased need for rescue analgesia, improved gastrointestinal recovery, earlier oral intake, and fewer opioid-related adverse effects. These findings support the integration of multimodal anesthesia as a core component of ERAS programs in gynecologic oncology and highlight its potential to enhance perioperative outcomes without compromising patient safety.
